# Plant Age and Soil Texture Rather Than the Presence of Root Hairs Cause Differences in Maize Resource Allocation and Root Gene Expression in the Field

**DOI:** 10.3390/plants11212883

**Published:** 2022-10-28

**Authors:** Minh Ganther, Eva Lippold, Manuela Désirée Bienert, Marie-Lara Bouffaud, Mario Bauer, Louis Baumann, Gerd Patrick Bienert, Doris Vetterlein, Anna Heintz-Buschart, Mika Tapio Tarkka

**Affiliations:** 1 Helmholtz Centre for Environmental Research, Theodor-Lieser-Str. 4, 06120 Halle, Germany; 2 TUM School of Life Sciences, Technical University of Munich, Alte Akademie 12, 85354 Freising, Germany; 3 Helmholtz Centre for Environmental Research, Permoserstr. 15, 04318 Leipzig, Germany; 4 Institute of Agricultural and Nutritional Sciences, Martin Luther University Halle-Wittenberg, Von-Seckendorff-Platz 3, 06120 Halle/Saale, Germany; 5 Swammerdam Institute for Life Sciences, University of Amsterdam, Science Park 904, 1098 XH Amsterdam, The Netherlands; 6 German Centre for Integrative Biodiversity Research (iDiv) Halle-Jena-Leipzig, Deutscher Platz 5e, 04103 Leipzig, Germany

**Keywords:** nutrient availability, water availability, *Zea mays*, rhizosphere, development, soil

## Abstract

Understanding the biological roles of root hairs is key to projecting their contributions to plant growth and to assess their relevance for plant breeding. The objective of this study was to assess the importance of root hairs for maize nutrition, carbon allocation and root gene expression in a field experiment. Applying wild type and root hairless *rth3* maize grown on loam and sand, we examined the period of growth including 4-leaf, 9-leaf and tassel emergence stages, accompanied with a low precipitation rate. *rth3* maize had lower shoot growth and lower total amounts of mineral nutrients than wild type, but the concentrations of mineral elements, root gene expression, or carbon allocation were largely unchanged. For these parameters, growth stage accounted for the main differences, followed by substrate. Substrate-related changes were pronounced during tassel emergence, where the concentrations of several elements in leaves as well as cell wall formation-related root gene expression and C allocation decreased. In conclusion, the presence of root hairs stimulated maize shoot growth and total nutrient uptake, but other parameters were more impacted by growth stage and soil texture. Further research should relate root hair functioning to the observed losses in maize productivity and growth efficiency.

## 1. Introduction

Maize is an important nutritional food source for millions of people and animals [1], but intensive maize cultivation requires considerable amounts of resources including agrochemicals, water, and mineral nutrients, and is therefore vulnerable to changes in soil quality [2,3]. Efforts to optimize maize production have led to an increased interest in root traits at the levels of root architecture and root hair formation [4], as well as physiological features of roots such as root exudation, nutrient and water uptake [5,6]. 

Plant growth is dependent on the availability of resources that can be allocated to growing tissues to support resource-demanding processes of tissue formation and cell growth. Control of photosynthetic rate and C allocation is as important during this process as the availability of nutrients and water in soil, and their uptake and allocation to the active meristems [7,8,9]. When resources are available, the plant has to balance their allocation between growth, maintenance and defense against detrimental organisms [10,11]. Therefore, resource allocation studies can indirectly reveal how the plant integrates the information from abiotic and biotic environments. Among plant organs, the root represents a particularly influential sink that additionally regulates not only sink-source interactions in the shoot, but also between the roots and rhizosphere organisms [11,12]. 

Greenhouse experiments showed that adaptation of the architecture of maize root system is an important prerequisite for nutrient and water uptake [13]. Maize produces a highly branched root system, composed of multiple root types formed at different developmental stages [14]. Lateral roots of maize play a major role in soil exploration and foraging for nutrients and water [15]. Their architecture is central for these traits: For instance, reduced frequency of lateral root branching stimulates not only N acquisition from low-N soils [16], but also drought tolerance [17] in maize. Aging of maize roots also affects their functional potential, such as changes in developmental and physiological processes relevant for growth, differentiation and stress tolerance [18,19]. 

Root hairs are specialized tubular outgrowths of single epidermal cells. Their formation dramatically increases the volume of exploited soil, and they are attributed major roles in mineral nutrient uptake as well as in beneficial interactions with soil microorganisms [20]. Despite the well-documented role of root hairs in mineral nutrient uptake [21,22], their role in water uptake remains controversial, and their quantitative contribution for individual elements is not well understood [21,23]. Root hairs had only a minor contribution to soil-plant hydraulics [5], and this effect was also less prominent in maize than in barley [24]. [25] investigated barley genotypes with differential capabilities for root hair formation under contrasting soils and climate conditions in a field experiment. Under well-watered conditions, root hairs had no beneficial effect on barley growth, but conferred a notable advantage under drought, with enhanced plant water status, P accumulation, and yield. This underlines the importance of field studies under variable environmental conditions to disentangle the role of root hair formation in stress responses.

Relationships between soil and plant mineral composition are element-specific and are strongly influenced by the environment [26]. Leaf mineral composition, the leaf ionome, reflects the complex interaction between a plant and its environment including substrate properties, fertilization and soil moisture, influential factors that can limit plant productivity. By contrast, total nutrient contents can be used to estimate total nutrient uptake of the plant. Importantly, a deficiency in a single nutrient may change the mineral balance as it is cross-related to the levels of other nutrients [27]. Elemental composition of a plant is affected by the availability of elements in the environment, root exudation-based solubilization and uptake of the elements, transport processes, plant metabolism and the requirements of each element [28,29]. Experimental and field data indicate that mycorrhiza formation is also important for maize P nutrition [30,31]. The expression and activity of nutrient and water uptake systems can be regulated locally in response to substrate availability, and systemically, by feedback control exerted by the plant signaling of the systemic nutrient status [32]. The plasticity of the root system may also play a role; e.g. in order to optimize their N nutrition, plants are able to direct C allocation [33] and enhance root growth in nitrate-rich patches [34]. 

The objective of this research was to assess the importance of root hair formation, substrate and plant development on the nutrition and root gene expression of field-grown maize [35,36]. Our earlier experiments in soil column experiments showed that maize roots were longer and thinner in loam than sand substrates, irrespective of the presence of root hairs [37]. Soil texture rather than root hairs dominated water uptake and soil-plant hydraulics [5]. Gene expression analysis also showed a much stronger effect for substrate than for root hairs, and e.g. gene functions related to immunity, stress, growth and water uptake were differentially expressed between sand and loam, and in a soil depth-related manner [38]. The general changes in the field regarding root architecture and water relations on the same experimental platform as this study were recently reported [39]. Adding to that, it was shown that soil substrate had a larger impact on P solubilization in the rhizosphere of maize than the presence of root hairs [40]. 

In this study, we surveyed a growth season of maize at three time points, comparing maize wild type to the root hairless mutant *rth3* grown on two substrates (loam, sand) with differing nutrient mobilities. The work was directed by four hypotheses. As plants have to finely balance their resource allocation to service growth, maintenance and defense processes [11], we first expected that C allocation in the root is at its highest in young seedlings, and that it positively correlates with growth and leaf nutrient concentrations. Following this, we expected pronounced stress-related gene expression in older seedlings. Our second hypothesis stated that there is an increasing effect by substrate during the aging of maize due to nutrient limitation and emerging water stress at the later developmental stages [41] (Supplementary Material Figure S1). We expected that the substrate effect is characterized by lower leaf nutrient concentrations [42,43] and higher nutrient transporter levels in sand than in loam, as topsoil drying is more detrimental for nutrient availability in sand. Since the topsoil is more subjected to drying than deeper soil, the third hypothesis stated that the effect of soil depth becomes more pronounced throughout the growing season. And finally, as the fourth hypothesis we expected that the subtle effects by root hair formation on root traits, water uptake and root gene expression [5,37,38] are exacerbated in the field, but qualitatively cause similar changes in gene expression than those observed in the previous laboratory experiments. 

## 2. Results

### 2.1. Maize Growth, Allocation of Recently Assimilated Carbon and Growth-Related Gene Expression

**Figure 1:**
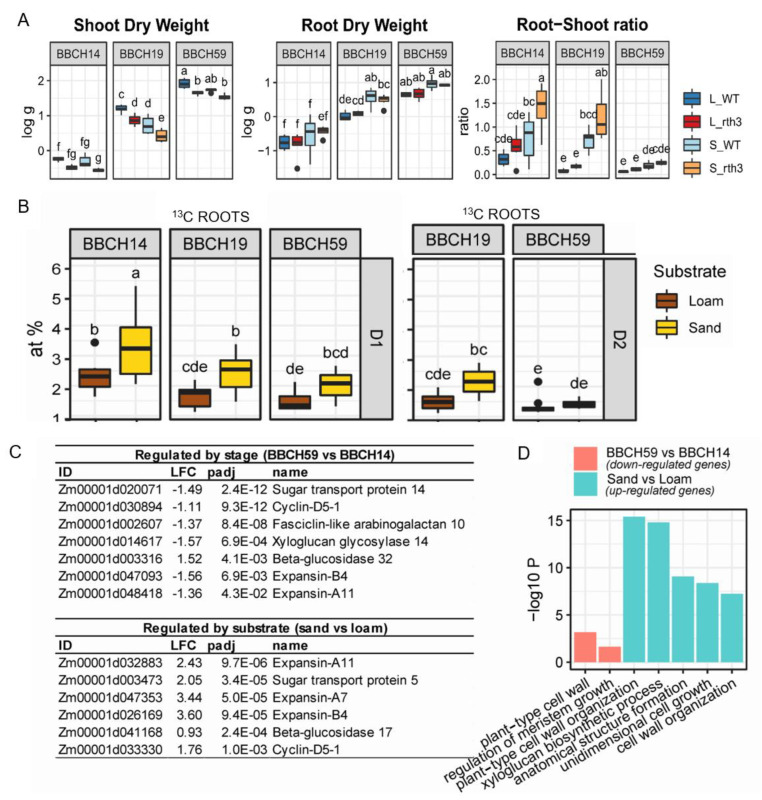
Root-shoot parameters, recently incorporated root carbon (^13^C atomic %), and growth-related root gene expression in maize. (**A**) Shoot and root biomass (log scale) and root-shoot ratio. L: loam, S: sand, WT: wild type, RTH3: root hairless maize. (**B**) C allocation (atomic %) to roots (D1: depth 0–20 cm; D2: depth 20–40 cm). Differences between treatments (*p* < 0.05) across stages according to ANOVA and Tukey’s HSD test are indicated by different letters. The presence of root hairs on C allocation was not significant. (**C**) Root gene expression related to cell wall expansion (contrast top: BBCH59 vs. BBCH14, bottom: sand vs loam; LFC: log2 fold change, padj: Benjamini Hochberg adjusted *p*-value). (**D**) Significantly enriched gene ontology terms related to cell wall expansion and growth (-log10P: -log10 *p*-value of the enrichment).

The root hair-deficient *rth3* displayed similar root dry weights to the wild type, but the shoot dry weights were approximately 50% lower (ANOVA and Tukey-Test, *p* < 0.05) during the three growth stages, leading to 50% higher root-shoot ratios in sand for BBCH14 and BBCH19 (Figure 1A). Maize shoot dry weight was 30% higher in loam compared to sand during BBCH19 (Figure 1A). For root dry weight, the opposite trend was observed: higher values (approximately 30%) were found in sand. Accordingly, root-shoot ratios were higher in sand and pronounced during BBCH19 (Figure 1A). 

The allocation of recently assimilated carbon (C) to shoots was not affected by the presence of root hairs, but it decreased from BBCH14 to BBCH59 (Supplementary Material Figure S2). Similar pattern was observed in roots: C allocation decreased over time, and it was not affected by the presence of root hairs. Furthermore, the amount of C allocation was higher for sand compared to loam at BBCH14 in shoots and at BBCH14 as well as BBCH19 in roots (Supplementary Material Figure S2 and Figure 1B). Substrate based difference was significant in the roots from the depth 1 (0–20 cm), but not from the depth 2 (20–40 cm). Gene expression analysis of roots between BBCH59 and the earlier stages of growth revealed that several cell wall and growth-related genes were down-regulated, including *beta-glucosidase 40*, *xyloglucan endotransglucosylases*, *expansins* and *fasciclin-like arabinogalactan proteins*. Similar genes were up-regulated in sand compared to loam (Figure 1C). These genes belonged to several enriched GO terms that also related to plant-type cell wall organization (Figure 1D). 

### 2.2. Effects of Growth Stage, Substrate and Genotype on the Elemental Concentrations of the Youngest Unfolded Leaf of Maize

**Figure 2:**
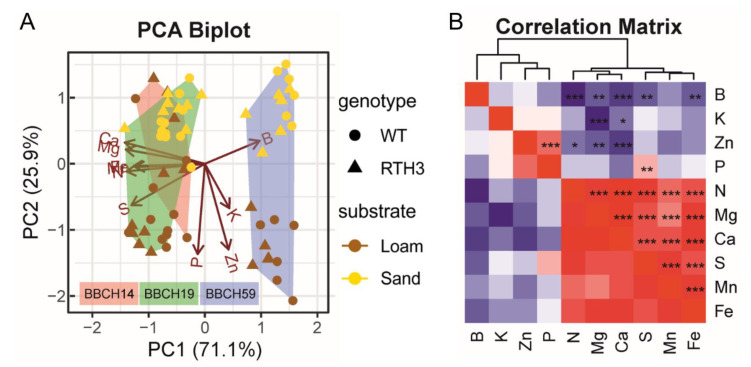
Developmental stage, substrate and maize genotype-dependent distributions of elemental concentrations in a young maize leaf. (**A**) PCA biplot visualization of elemental composition analysis of the macro- and micronutrient concentrations in the youngest fully developed maize leaf. The PCA plot combines the data from the three developmental stages (convex hulls). Substrate and genotype treatments are encoded as described in the legend. (**B**) The correlation matrix shows the results of correlation tests (Spearman correlation) between the concentrations of the individual elements, where color indicates negative (blue) or positive (red) correlations. Level of significance: *** *p* < 0.001, ** *p* < 0.01, * *p* < 0.05.

Next, we investigated the elemental concentrations in the youngest unfolded leaf (YL, momentary nutrition status). PERMANOVA indicated highly significant growth stage-dependent differences between the YL elemental concentrations (R^2^ = 0.541, *p* < 0.001) and a significant impact by soil substrate (R^2^ = 0.141, *p* < 0.001), but maize genotype (WT/*rth3*) had a minor impact (R^2^ = 0.012, *p* < 0.022). These observations were supported by principal component analysis (Figure 2A). When the datasets were partitioned according to the three growth stages, an increasing impact of substrate over time (i.e., from BBCH14 to BBCH59) became evident. 

**Figure 3:**
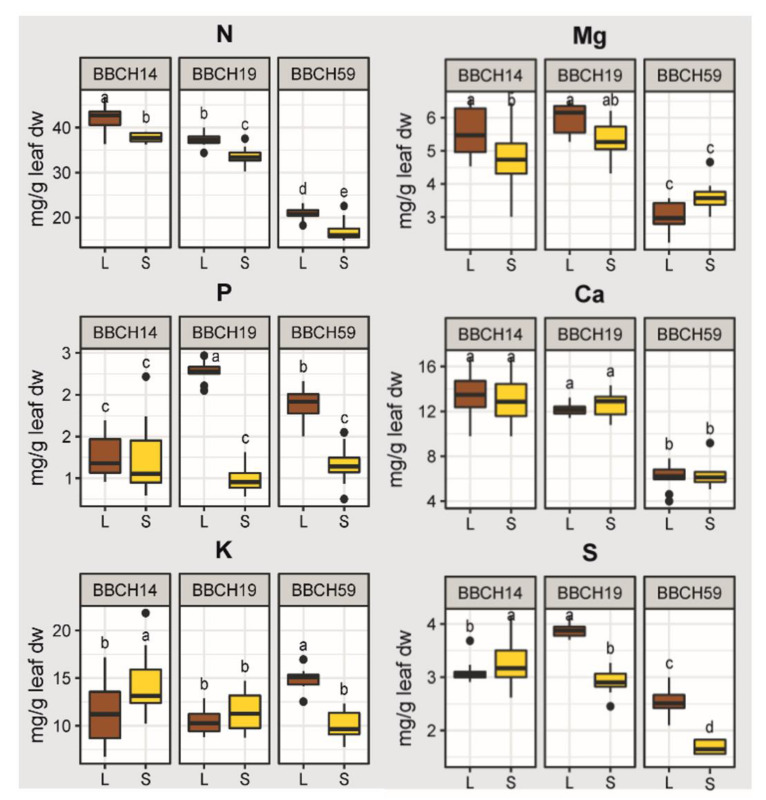
Macronutrient concentrations in young leaves over time and substrate. Leaf macroelement concentrations over time and in two different soils. Treatments with different letters are significantly different (ANOVA, Tukey’s HSD test, *p* < 0.05). L, loam; S, sand. The effects of genotype were not significant are thus not shown.

**Figure 4:**
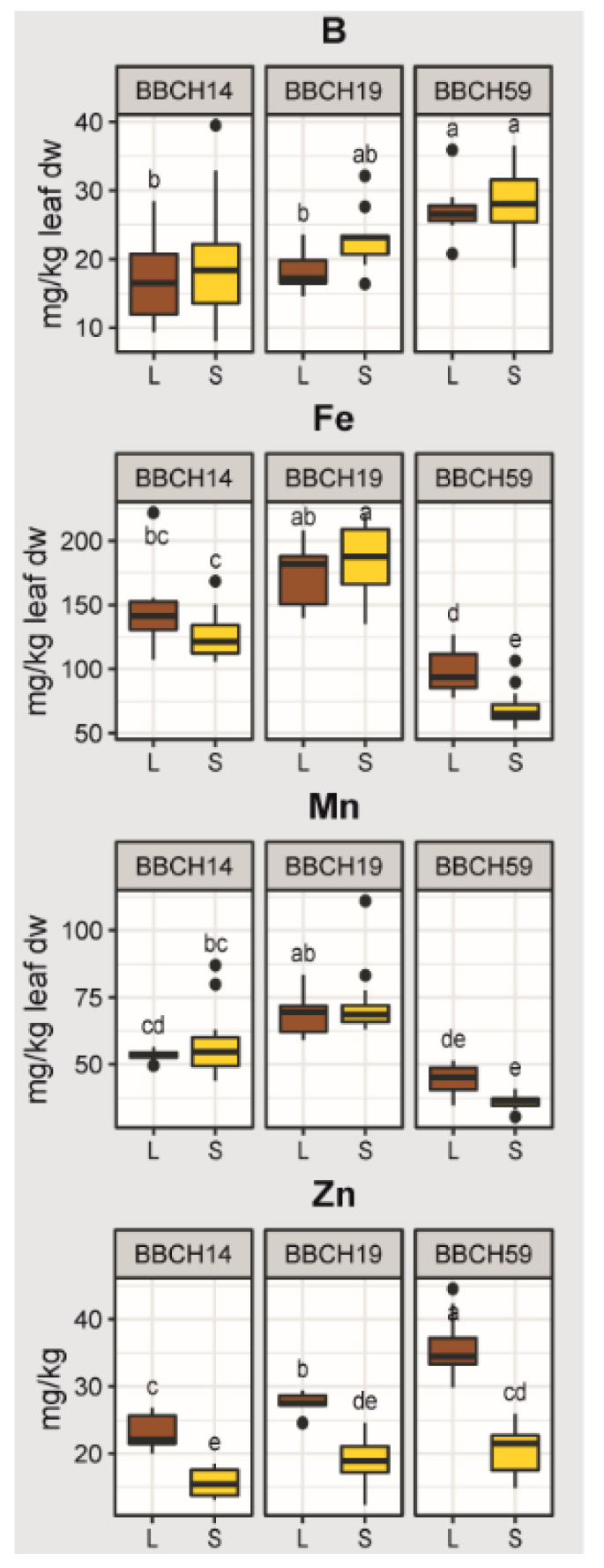
Micronutrient concentrations in young leaves over time and substrate. Leaf macroelement concentrations over time and in two different soils. Treatments with different letters are significantly different (ANOVA, Tukey’s HSD test, *p* < 0.05). L, loam; S, sand. The effects of genotype were not significant are thus not shown.

For YL elemental concentrations, we found no difference between substrates during BBCH14, but a high significance (R^2^ = 0.67, *p* < 0.001) during BBCH59. Apart from that, the influence of genotype on the ionome became significant (R^2^ = 0.086, *p* = 0.008) only during BBCH59. Significant positive correlations between YL elemental concentrations were detected between the concentrations of Zn and P, but also between N, Mg, Ca, S, Mn and Fe (Figure 2B). By contrast, negative correlations occurred between B and N, Fe and Ca, as well as K and Mg, but also between Zn and Ca. These results demonstrated that changes elemental concentrations of the YL are partly explained by growth stage and by substrate and that the presence of root hairs has only a minor influence. With the macronutrients N, Ca, Mg, and S in young leaves, we saw a general decline of leaf elemental concentrations over time while comparing the first two stages with the last stage BBCH59, and the N concentrations were higher in loam than sand (Figure 3). Ca, Mg and N concentrations in young leaves were relatively constant at BBCH14 and BBCH19, followed by a drop at BBCH59. The concentration of P and K remained stable during maize development, but varied by substrate during specific stages of growth. P concentrations were considerably higher in loam than sand during BBCH19 and BBCH59 (Figure 3). The concentration of K was higher in sand than loam during BBCH14, but higher Mg concentrations were found for loam during BBCH14. By contrast, Ca element concentrations were not affected by substrate. For S, substrate had no effect during BBCH14, but at later stages higher concentrations were found in loam than sand. Microelement levels in young leaves changed in an element-related pattern with time and substrate (Figure 4). Concentrations of Zn and B increased with time; additionally, Zn concentrations were higher in loam than sand. Fe and Mn concentrations also exhibited a temporal pattern. For these elements, the concentrations rose during BBCH19, but decreased during BBCH59, even below the initial level during BBCH14. Substrate-dependent differences could be identified for Fe during BBCH59; higher in loam than sand (Figure 4). To sum up, based on the nutrient concentrations of young leaves, supply of P, K and Zn during the three growth stages and supply of N and Mn during BBCH59 were below the level classified as adequate for maize growth, according to Bergmann (1986) [44].

### 2.3. Elemental Concentrations in the Remaining Shoot

The changes in YL mineral element concentrations were also present in the remaining shoot (RS, nutrient concentration). In concordance with the data from YL, growth stage (permutational multivariate ANOVA; R^2^ = 0.48, *p* < 0.001) and substrate (R^2^ = 0.135, *p* < 0.001) exerted strong effects on RS elemental concentrations, while root hair genotype had a lower impact (R^2^ = 0.044, *p* < 0.001; Supplementary Material Figure S3; Table S1). As described for young leaves, the substrate effect strongly interacted with the growth stage (R^2^ = 0.09, *p* < 0.001; Figure S4). Since the supply of N, P, K and Zn was low according to Bergmann (1986) [44], and these mineral nutrients can be moved to areas of active growth within the plant during nutrient deficiency, we subsequently cross-compared their concentrations between the YL and the RS. During BBCH14, N concentration was higher in YL than RS, but K and Zn lower (Supplementary Material Figure S4). During BBCH19, N concentration was higher in YL than RS, but P, K and Zn lower. During BBCH59, N and K concentrations were higher in YL than RS.

### 2.4. Incorporation of Mineral Elements to Maize Shoot Biomass

**Figure 5:**
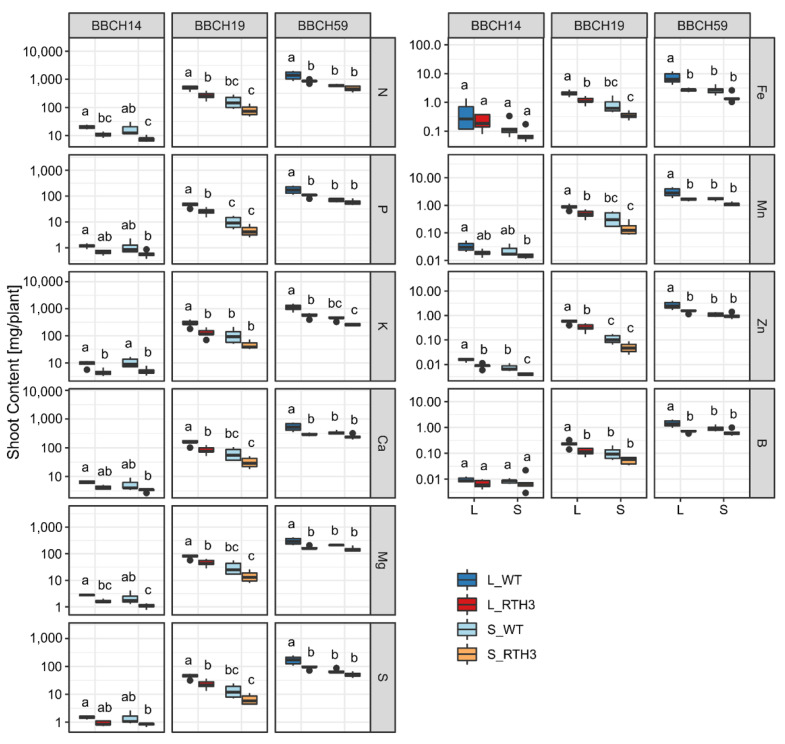
Total mineral element contents (mg per plant) of the shoots. The amounts of the mineral nutrients are given by mg/plant. Note the different scales for the different mineral nutrients. Differences between treatments (*p* < 0.05) within one stage according to ANOVA and Tukey’s HSD test are indicated by different letters. L, loam: S, sand; WT, wild type; RTH3, root hair mutant.

Considering that shoot dry weights were consistently lower in *rth3* than wild type (Figure 1A), we performed an analysis on the total amounts of elements (mg Ca, K, Mg, N, P, S, B, Fe, Mn and Zn) in shoots, essentially representing the final product of mineral nutrient concentrations and shoot dry weights. According to PERMANOVA, the whole shoot elemental contents were moderately influenced by both the substrate (R^2^ = 0.080, *p* < 0.001) and the genotype (R^2^ = 0.044, *p* < 0.001); these effects interacted with growth stage (R^2^ = 0.064, *p* < 0.001 stage * substrate; R^2^ = 0.041, *p* < 0.001 stage * genotype). The total amounts of elements were in general higher in loam than sand, and in several samples higher in wild type than *rth3* maize (Figure 5; Supplementary Material Table S2). Precisely, the total N, K, Mg, Fe, and Zn amounts were higher in WT than in *rth3* in 4 of the 6 treatments, and the total P, Ca, S, Mn and B amounts in in 2 of the 6 treatments (Figure 5). The influence by root hairs was stronger during BBCH19 and BBCH59 than BBCH14 for Ca, S, B, Fe and Mn, and the effect by substrate was stronger during the later growth stages for all elements except for Zn (Figure 5). 

### 2.5. The Expression of Root Nutrient Transporter Genes

Substrate and growth stage had an impact on the expression of nutrient uptake transporters and acid phosphatases in maize roots (Supplementary Material Figure S5; Table S3). Specifically, during BBCH59, *High affinity N transporter* and *NRT2*, but also sulphate transporter *SULTR1.2* and Zn transporter *ZIP2* were upregulated during BBCH59 in sand, K transporters *HAK2* and *HAK4* in loam, and boron transporters *RTE* and *RTE2* in both substrates. High affinity P transporters *PHT1* and *PHT2* were more highly expressed during BBCH19 and BBCH59 than BBCH14. By contrast, several purple acid phosphatase genes were up-regulated during BBCH14 (e.g., *PAP7c*/*d*, *PAP10*, *PAP13*, *PAP16*, *PAP23*) and four genes showed higher expression in sand than loam during BBCH19 and BBCH59 (e.g., *PAP1c*, *PAP7c*/*d*, *PAP10*, *PAP13*) (Table S3). 

### 2.6. General Enrichment Patterns of Differentially Expressed Genes (DEGs) in Maize Roots

**Figure 6:**
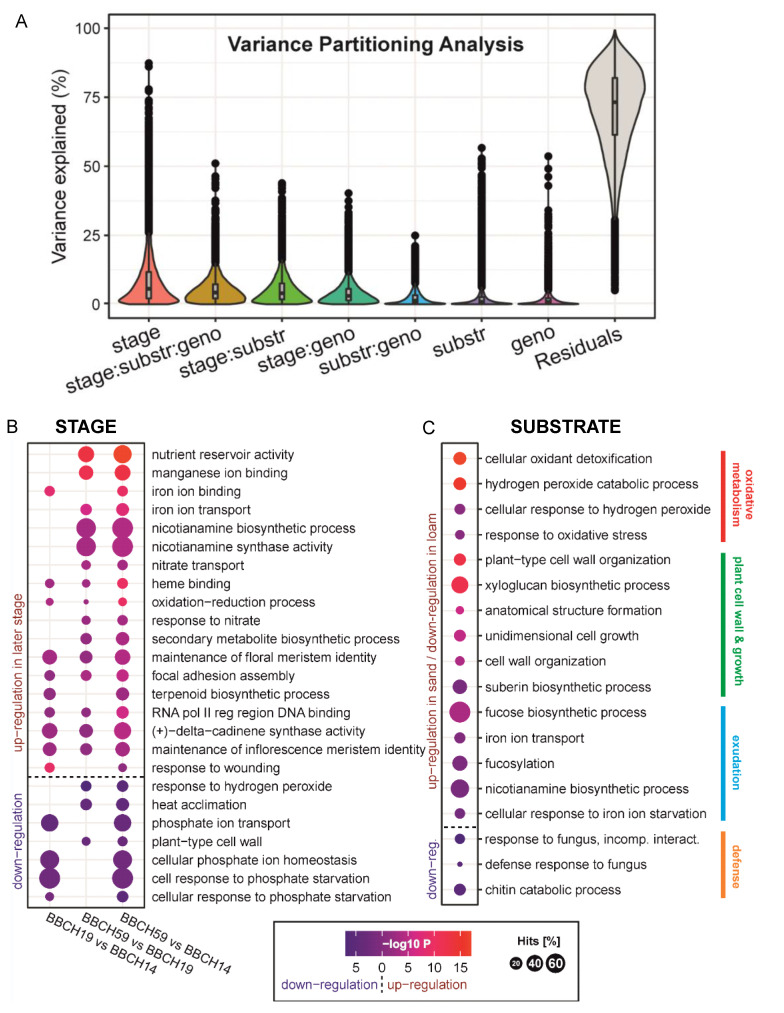
Root gene expression and the enrichment of Gene Ontology terms according to growth stage and substrate. (**A**) Variance Partitioning Analysis of RNAseq data with explained gene variance attributed to each experimental factor. (**B**), (**C**) Gene Ontology (GO) terms enriched by up- or down-regulated genes (*p* < 0.05) in the roots of maize. Point size encodes enrichment level (coverage or hits of differentially expressed genes within a specified GO category). (**B**) Effect of growth stage in the comparisons between BBCH19/BBCH14 (left panel), BBCH59/BBCH19 (middle), and BBCH59/BBCH14 (right). Red indicates up-regulation in later stages, blue up-regulation in earlier stages. (**C**) Effect of substrate (sand/loam). Red indicates up-regulation in sand, blue up-regulation in loam.

The profiling of global maize root gene expression accompanied with variance partitioning analysis (Figure 6A) identified highly significant growth stage and soil substrate dependent variation, but a low effect by maize genotype. 

The total DEG numbers between the treatments are listed in Supplementary Material Table S4. A more detailed list of differentially expressed genes with log_2_ fold changes and adjusted *p*-Values between the treatments of interest is provided in Supplementary Material Table S5. The identified DEGs were surveyed for common gene functions by gene set enrichment analysis by assigning gene ontology (GO) descriptors (Supplementary Material Table S6). For the genes that increased in expression over maize development from BBCH14 to BBCH59 (Figure 6B), the overrepresented GO terms included plant nutrition related *manganese ion binding*, *iron* and *nitrate transport*, and *nicotianamine synthase activity*, but also genes related to secondary metabolism, i.e., *terpenoid biosynthesis*. In contrast to this, genes that decreased over time led to an enrichment of GO terms such as *plant type cell wall* and *phosphate ion transport*. Differential gene expression by substrate (Figure 6C) with combined three growth stages data was reflected by overrepresented GO terms in sand related to oxidative stress including *cellular oxidant detoxification*, but also cell wall organization and biosynthesis-related terms including *xyloglucan* and *suberin biosynthetic process,* and iron and zinc nutrition related *nicotianamine synthase activity*. Conversely, GO terms enriched by DEGs with a higher expression in loam were related to defense responses against fungi (e.g., response to fungus, chitin catabolic process). Since the growing season was characterized by low level of precipitation (Supplementary Material Figure S1), we specifically addressed how plasma membrane aquaporin genes are expressed in maize roots. Aquaporin gene expression was higher in sand compared to loam, and increased substantially over time (Figure S6; Table S3). It was not affected by root hair formation.

### 2.7. Maize Root Gene Expression in Two Depths of the Soil

To assess the impact of soil depth on maize root gene expression, roots were sampled from two depths (D1 0–20 cm, D2 20–40 cm) and the expression of a subset of 41 genes involved in plant defense, stress, exudation, and nutrient transport was analyzed by real-time qPCR. Depth profiles were sampled during BBCH19 and BBCH59, as plants were not yet rooted to the second depth during BBCH14. Calculated log_2_ fold changes derived from qPCR array and RNAseq data of the upper depth were in accordance with each other (Supplementary Material Figure S7). Similarly, as described for RNAseq analysis and shoot element composition, growth stage was generally the strongest impact factor on gene expression profiles, followed by substrate (PERMANOVA, *p* < 0.001 and *p* = 0.002, respectively). For qRT-PCR data, with progressing time the differences between sand and loam expression profiles became more pronounced, and were at their highest during BBCH59. The same trend was true for depth profiles. Although not overall significant (PERMANOVA, *p* = 0.117), we found that 15 of the surveyed 41 genes were differentially expressed at BBCH59 in loam, and 8 in sand (Supplementary Material Table S7). DEGs with higher expression in D1 (0–20 cm) compared to D2 (20–40 cm) regardless of substrate were aquaporins (*PIP1-5*, *PIP2–3 and PIP2–6*), defense-related *NPR1, disease resistance RPP-like 4* and *jasmonate-induced protein*, as well as exudation-related *Al-activated malate transporter* and *transporter of mugineic acid*. The K transporter *ZORK* showed higher expression in the bottom layer D2. While the depth-related DEG in sand were almost all present in loam as well, some N and P transporter genes showed differential expression by depth specifically in loam: Upregulated in D2 in loam were the ammonium transporters *AMT2*, *AMT7* and *NRT2*, and the P transporters *PHT2* and *PHT12*.

### 2.8. The Influence of the Presence of Root Hairs on Maize Root Gene Expression

Only 139 genes (*p* < 0.05, |LFC| > 0) were differentially expressed between *rth3* and WT among the three growth stages, which supported the low significance level of the PERMANOVA results. About 20% (23 genes) of these DEGs were previously detected as differentially expressed by maize genotype in laboratory experiments of young primary root gene expression (Rüger, MG, in preparation) or BBCH14 stage root system [38] (Supplementary Material Figure S8; Table S8).

## 3. Discussion

### 3.1. Development, Soil Texture and Drought Drive Maize Gene Expression and Nutritional Status, While the Presence of Root Hairs Supports Shoot Growth

The growth stage was a decisive driver for elemental composition of maize leaves, since the macroelements N, Ca, Mg and S, but also the microelements Zn, B, Fe and Mn exhibited a temporal pattern. Maize development also represented a moderate driver of root gene expression, with e. g. increased manganese and iron ion binding, as well as terpenoid biosynthesis related gene expression during the stage of tassel emergence. For both element composition and gene expression, the effect of substrate is enhanced during the aging of maize, and the development of drought stress. These results provide experimental evidence how maize responds to the environment, and support the aim to increase the sustainability of maize cropping systems [45]. The presence of root hairs mattered for shoot growth and total nutrient accumulation, but had a minimal influence on element concentrations and root gene expression profiles. This suggests that the root hairs are important for efficient nutrient acquisition and maize growth propagation [46]. 

### 3.2. Lack of Root Hairs Is Reflected by a Small Set of Up-Regulated Genes in the Roots but a Substantial Negative Impact on Maize Shoot Growth

We observed that the global gene expression patterns were highly similar between *rth3* and WT maize, although the loss of elongated root hairs resulted in lower shoot biomass and lower total uptake of nutrients. This observation opposed part our fourth hypothesis, which stated that effects of root hair formation on root traits and root gene expression should be higher in the field than laboratory settings. The minor extent of changes in gene expression by genotype was, however, highly consistent with earlier data on maize root architecture [38] and root gene expression during BBCH14 in soil columns [39], and also regarding the transcript abundances in young primary roots (Rüger, MG, in preparation). The fact that the total uptake of nutrients was higher in WT, but not root biomass, or root architecture [39], might indicate the nutrient uptake and/or transport rates per root volume are higher in WT than *rth3*. Since transporter gene expression was not affected, it is possible that transporter activity is regulated at the protein level. For instance, post-translational modifications such as ubiquitination and phosphorylation are widespread among the members of phosphate transporter 1 family, affecting the localization, abundance and activity of the transporters [47]. Our data for total nutrient uptake were also in accordance with observations made in a greenhouse experiment assaying the *rth2* maize mutant which forms extremely short root hairs [48]: While the concentration of P did not differ in *rth2* compared to the wild type, the total P content of the juvenile maize plants was reduced by 50% under combined water stress and P deficiency, in agreement with the substantial importance of root hairs for P and water uptake in maize under low availability. Furthermore, WT plants did not show higher P concentrations, but higher total shoot P contents, a product of P uptake and biomass generation. Here, we observed a similar effect for a number of macro-and micronutrients (Figure 3 and Figure 4). Lower biomass gain by *rth3* could thus be related to a more inefficient use of the incorporated carbon by the root hair mutant. This could be caused for instance by higher respiration rate [49]. Alternatively, the root hairs might influence leaf water potential dependent growth. Namely, when cumulative water extraction was normalized to shoot dry weight, Jorda et al. (2022) detected higher water use efficiency of WT than *rth3* maize [41]. Lower water use efficiency of *rth3* could thus be explained by a more effective regulation of stomata in response to drought [50], which could also affect respiration rates. Another option is, that differences in plant hormone levels were involved in the observed differences of water use efficiency or shoot dry weight instead a higher respiration rate. Unfortunately, we have not measured if *rth3* has differences in the endogenous hormonal balance as compared to WT. Nevertheless, the upregulation ethylene biosynthesis enzyme gene *1-aminocyclopropane-1-carboxylic acid oxidase* and two *ethylene responsive transcription factor* genes in *rth3* as compared to WT warrants such investigations.

As expected, the *rth3* mutation caused qualitatively similar changes in gene expression to those observed in the previous laboratory experiments. In fact, 23 differentially expressed genes between *rth3* and WT (~20%) from this study have also been observed in two previous laboratory experiments. The effect (in terms of log fold change in expression) of root hair formation on this core gene set was consistent in these experiments, even though experimental size scales differed (soil column experiment: [38]; rhizobox experiment: Rüger, MG, in preparation). These results confirmed the second part of the fourth hypothesis, stating that qualitatively, *rth3* mutation causes similar changes in gene expression than those observed in laboratory experiments. Several of the genes in this core gene set point to cell wall-associated functions, such as *COBRA-like protein 7*, *xyloglucan 6-xylosyltransferase*, *tetraspanin18*, *beta-glucosidase aggregating factor1*, and *putative wall-associated receptor protein kinase*. Out of them, the COBRA-like protein gene is particularly interesting, since the *rth3* gene also encodes a COBRA-like protein [51]. COBRA proteins are involved in cell wall development across angiosperms, and members of the COBRA family act in cellulose formation and orientation [52]. The down-regulation of maize *COBRA-like 7* gene might thus be indicative of the *rth3* mutation-related changes in cell wall development which appears to remain stable from external influences. 

### 3.3. Growth Stage and Substrate as Drivers of Maize Nutrition

We found that maize plants did not receive adequate P, K and Zn from soil during the three growth stages, and were supplied with too little N and Mn during BBCH59 [44]. These limiting nutrient in part resulted from the design of the field experiment: To push the maize plant to invest into resource acquisition, the field experiment aims at achieving nutrient levels that are in the range between slightly nutrient deficient to adequate nutrition for the WT maize [35]. For BBCH59, the limitation of nutrients was particularly strong, most likely due to the fact that the topsoil dried out completely, rendering fertilizer-applied nutrients unavailable for plant uptake [53]. This observation was according to our second hypothesis, stating that there is an increasing effect by substrate during the aging of maize. Low water availability and high temperatures could have also limited shoot growth [54,55] and thus decreased nutrient demand. Leaf [56,57] and root [58] ionome studies from hydroponics and from the field [59] have shown that the mineral nutrient homeostasis is mediated by strong interactions between individual nutrients. In line with this, our field study found significant correlations between the concentration profiles; the macronutrients N, Ca, Mg and S, and micronutrients Fe and Mn followed a similar trend (Figure 2B). The reports on interactions between multiple nutrient elements suggest that their uptake, transport, and/or assimilation levels are correlated, and supports chemical element stoichiometry in plant tissues [60]. For instance, Briat et al. (2015) presented a molecular framework in *Arabidopsis thaliana* for interactions between P, S, Fe and Zn homeostasis [61]. In our study, these nutrient elements showed in part contrasting patterns according to sampling stage and substrate (Figure 3 and Figure 4), suggesting that the nutrient levels were not balanced. 

During the transition between vegetative and reproductive growth [62], but also when root mineral uptake is limited and cannot fully support plant growth [63], nutrient reallocation has been identified as a strategy by higher plants to improve mineral resource availability of sink tissues. Whereas N can be readily remobilized from leaves, K, S, P, Mg, Cu, Mo, Fe and Zn are predominantly mobilized during nutrient deficiency [58]. Our data showed lower N and Mn levels in remainder shoot (RS) than younger leaves at all stages, and lower K levels during BBCH59 (Supplementary Material Figure S4). Leaf mineral nutrient remobilization has been described in maize, but maize was among the least efficient plants in nutrient reallocation from senescent to young leaves [63]. Our data thus indicates that maize did not efficiently compensate for the loss of nutrients in young leaves by nutrient reallocation from the rest of the shoot. 

### 3.4. Decreased Element Concentrations in the Shoot and the Activation of Nutrient Transporters in the Roots

Nutrient starvation cues are immediately transmitted from the shoots to the roots via signaling molecules to maintain nutrient homeostasis. This signaling may lead to the activation of nutrient uptake transporter gene expression in roots, secretion of organic acids, siderophores or enzymes, associations with mycorrhizal fungi and alterations in root architecture and growth to enhance mineral uptake [64]. This implies that low levels of nutrients in the shoot may lead to higher expression of nutrient transporters in roots; such relationships were observed in this study. High-affinity transporters *NRT1* and *NRT2*, that are induced by N starvation [65], were upregulated in sand, the substrate that was associated with a lower N concentration of shoot. A similar pattern was found for P transporters *PHT1* and *PHT2*, that was in line with Yu et al. (2018), who observed that transcript accumulation of all *PHT1* genes is negatively correlated with P availability [30]. Furthermore, several purple acid phosphatases (Supplementary Material Table S3) that are known to be induced during P starvation [66], and the shoot P concentration, were up-regulated in sand. Interestingly, the data on acid phosphatase gene expression are in line with rhizosphere activity: Bilyera et al. (2022) observed that the average rhizosphere volume for acid phosphatase activity was greater in sand than in loam [40].

The stronger expression in sand than loam suggests that the topsoil in sand was dry and nutrient availability decreased, leading to higher mineral transporter gene expression. This hypothesis should be tested by cross-comparing the levels of available nutrients in soil and transcript abundancies of root transporter genes. The characterization of these gene “responders” in the field could be applied for future studies to target and possibly elicit certain plant responses to environmental stresses. Related to iron and zinc uptake by maize roots, one of the most highly enriched GO terms by developmental stage was *nicotianamine biosynthesis*. Nicotianamine represents a critical metabolite in the biosynthetic pathway to mugineic acid family phytosiderophores, natural Fe and Zn chelators [67], that graminaceous plants secrete from their roots to mobilize and take up Fe and Zn from the rhizosphere [68,69,70]. As Fe concentration in the maize leaf remained low during BBCH59, the elevated expression of genes involved in nicotianamine biosynthesis could be interpreted as a response to that. 

### 3.5. Young Maize Plants with Higher Carbon Assimilation and Growth Related Gene Expression Rates

Root resource allocation and biomass partitioning were also affected by plant age, with a higher level of carbon (atomic % of ^13^C) uptake during BBCH14, according to our first hypothesis, and higher root-shoot ratio during BBCH14 than BBCH59, in both substrates (Figure 1A,B). This suggests a relative increase in shoot sink activity at the cost of the root system, which would be normal during maize development in soils under no water limitation. Since the precipitation was low during BBCH19 and continued to be so during BBCH59 (Supplementary Material Figure S1), a stronger resource and biomass investment to the roots than during sufficient soil moisture was expected [71]. Root sink limitation by environmental factors, like water or nutrient availability, may thus be more constraining for growth and development than C availability [7]. The temporal pattern of root resource allocation was also reflected in growth related gene expression. When root C allocation decreased over time, this was accompanied by a decrease in cell wall-related gene expression (e.g., *beta-glucosidase 40, xyloglucan endotransglycosylase*). Comparing substrates, C allocation was higher in sand than loam (Figure 1B and Supplementary Material Figure S2). In terms of gene expression, this was supported by a higher gene expression in sand for transport and cell wall-related genes (Figure 1D). Wu et al. (2001) observed that gene-specific regulation of *α-* and *β-expansins* likely contributes to growth alterations of the maize root [72], but expansin gene expression was not markedly altered in this study, neither by development nor substrate. Our findings were in line with higher root length densities of plants grown on sand [39]. Root length density (root length per volume soil) describes root architecture in respect to the size of the soil volume that can be explored by roots, and it is thus related to the efficiency of water and nutrient uptake [73]. By contrast, for plants on loam we found an enrichment of genes supporting GO categories related to microbial defenses and stress (for example chitin, resp. to herbivory; Supplementary Material Table S5). These findings support our first hypothesis, that the balancing of resources in order to favor either growth or defense [11] is reflected on a transcriptomic level.

### 3.6. Water Stress towards BBCH59 Leads to Differences between Substrates in Root Plasma Membrane Aquaporin Expression

Within the same field experiment, water transpiration data from Jorda et al. (2022) demonstrated that severe water stress occurred just before tassel emergence (BBCH59 stage) [41]. Water stress is especially detrimental during the reproductive stages when water requirement is the highest [74,75,76]. According to the water transpiration data, drought stress occurred earlier in loam than sand substrate and earlier in the wild type than the *rth3* mutant. This could be possibly attributed to the higher root-shoot ratios observed in plants grown on sand, as an adaptation to water stress [71]. In wheat, it was shown that increased root-shoot ratios supported growth under drought conditions, as they served to limit inefficient transpiration [77,78]. For maize, transpiration-limiting variants were also estimated to produce to higher yields under extreme conditions [79]. 

Water transport in plants is conveyed by intrinsic membrane proteins of the aquaporin family [80], and the role of aquaporins in root water uptake has been abundantly documented [81]. Plant aquaporin genes are regulated in a complex manner, for instance some up- and others down-regulated by drought, suggesting that the maintenance of water transport requires spatial regulation of aquaporin gene expression [82,83]. Elevated aquaporin expression, with higher values for sand than loam was observed at BBCH59 (Figure S6). This would fit the idea of a compensatory pattern in aquaporin expression, considering measurements of plant available water by Vetterlein et al. (2022), that demonstrate a sharp decline of plant available water during the transition from BBCH19 to BBCH59 [39]. At BBCH59, plants on loam (both root hair genotypes) had already experienced drought stress for 1–2 weeks, but plants on sand (wild type) only for 4–5 days. The fact that aquaporin expression is higher in sand even though they experienced later onset of drought than loam could perhaps indicate a differential stress response in relation to immediate or prolonged water stress exposition. Plants grown on sand were sampled during the recent onset of drought stress which led to elevated aquaporin gene expression. Another possibility could be that plants on sand generally expressed higher levels of aquaporin genes and thus were better equipped to face drought stress. Elevated aquaporin expression in sandy substrate was also found for a companion soil column experiment conducted under finely controlled growth and watering conditions [38], suggesting that the induction could also in part be related to the properties of the root system. Interestingly, both field and lab grown plants developed thicker root diameters in sand [37,39], and the thicker roots might compensate for potential increase in radial resistance by increased aquaporin expression [84].

### 3.7. Depth Profiles Are More Pronounced at BBCH59 Than BBCH19 and Affect Selected N, P and Aquaporin Genes

At BBCH59 compared to BBCH19, more depth-related gene expression among the surveyed genes has been detected, which is in line with our third hypothesis, stating that depth profiles become more pronounced towards later growth stages. At BBCH19, no gene expression differences between depths 0–20 and 20–40 cm were observed, but at BBCH59 a stronger induction of N, P and water transporters in the upper layer, in particular for the loam substrate, was shown. Root length density was higher in the top 20 cm than the lower depth at BBCH59 [39], suggesting that the roots in the upper layer may have exhausted some of the available nutrients. This possibly was exacerbated by the drying out of the topsoil. For maize under field conditions, Wiesler and Horst (1992) observed that nitrate uptake rate per unit root length generally increased with soil depth, most likely due to increased root growth [85]. The higher expression of aquaporin genes *PIP1-5*, *2*–*3* and *2–6* in the upper soil is probably related to lower soil moisture, and higher expression levels of root exudation-related *malate transporter 10* in the topsoil suggests that root exudation is also enhanced by low soil moisture [86]. Of the analyzed maize phosphate transporters, *PHT2* [87] and *PHT7* [88] are up-regulated by arbuscular mycorrhizal fungi, implying that these transporters might participate with other P transporters in mediating inorganic P absorption and/or transport by the mycorrhizal pathway. Mycorrhiza formation in this experiment was higher in the topsoil than in the lower depth [39], and this matched the higher expression *PHT2* and *PHT12* in the topsoil layer, but only for plants grown on loam. 

## 4. Materials and Methods

### 4.1. Experimental Design and Field Study Setup

The experimental design and setup of the soil plot experiment (SPE) at the research station in Bad Lauchstädt, Central Germany, is described in extensive detail in [36]. Details on plant shoot and root growth as well as total shoot nutrient content were provided by Vetterlein et al. (2022) [39]. The sampling described in this study was conducted in the months May to July in 2019. Briefly, the field experimental setup consists of a two-factorial, randomized block design with six replicates represented by individual plots. One plot was excluded from analysis due to unintended compaction during its setup. 

The investigated factors are substrate (loam, sand) and maize root hair genotype (*Zea mays* B73 wild type, *rth3*). The substrate *loam* was derived from haplic Phaeozem soil (from 0 to 50 cm depth) in Schladebach, Germany (51°18′31.41′′ N; 12°6′16.31′′ E). The substrate *sand* was obtained by repeated mixing and sieving of 16.7 % loam with quartz sand (550 t, WF 33, Quarzwerke Weferlingen, Germany). The maize *rth3* mutant is genetically highly homozygous, and displays defective elongation of root hairs compared to the B73 wild type (WT). In field trials, it has been characterized with an unaltered phenotype, but exhibited reduced crop yields that were 20–40% lower compared to the wild type [51]. 

Substrate was supposed to drive differences for water and nutrient transport, thus differences in nutrient availability were compensated by fertilization (detailed in [35]). Pre-trials in soil columns were run to calibrate fertilization amounts to achieve similar shoot growth rates for the wild type on both substrates. As a result, sand was fertilized with twice the amount of N, P, K, Mg and Ca than loam, and additionally provided with micronutrients. These ratios were applied to the field as well, using field-conventional fertilizers applied to the soil surface (in kg ha^−1^ for loam/sand: N 50/100, P 12/24, K 50/100, Mg 18/33, Ca 27/52, micronutrients 0/100). Sampling was conducted at three different time points which represent different developmental stages of maize [89]. BBCH14 (4 leaves unfolded) and BBCH19 (9 or more leaves unfolded) represent vegetative growth stages, BBCH59 (end of tassel emergence) denotes the transition from vegetative to generative growth. These stages were chosen to cross-compare the rapidly growing root systems with a more mature root system, combined with three different parts of the growing season: late spring (first week of June; mean daily temperature 20.6 °C), early summer (late June; mean daily temperature 23.7 °C) and mid-summer (mid-July; mean daily temperature 20.6 °C). The basic weather data during May, June and July 2019 are shown in Figure S1. The precipitation in 2019 was low compared to the long-term average for the region, and led to the emergence of drought stress as the growing season advanced. In particular, water deficit between BBCH 19 and 59 resulted in visible symptoms of drought stress, i.e., leaf rolling [39].

### 4.2. Stable Isotope Labeling and Root Sampling

The allocation pattern of recently assimilated carbon was assessed by stable isotope labeling. The application procedure of labeled ^13^CO is detailed in [35] which describes the experimental setup including the present study. Sampling was conducted between 10:00 and 16:00 h for a single C-labeled maize plant per plot. The shoot was cut off at the base and brought to the on-site laboratory for elemental analysis. For root harvest, 20 × 20 × 20 cm soil next to the maize plant was excavated and directly sampled (depth 0–20 cm, D1; all growth stages), followed by the excavation and direct sampling of the subsequent 20 × 20 × 20 cm soil of the second depth (depth 20–40 cm, D2; only for growth stages BBCH19 and BBCH59). Root growth parameters from a soil core (5 cm diameter) in the center of the 20 × 20 × 20 soil cube were provided by Vetterlein et al. [39]. From the remainder of the soil volume, all roots were manually sorted out. Attached soil was gently loosened with a soft toothbrush to avoid root damage. Roots were cut up with scissors and mixed to obtain a representative mixture of root types. An aliquot of 2–3 g roots (fresh weight) representing different root types was sampled from this mixture for root gene expression and C/N content analyses. The roots were submerged and vortexed 3 times for 10 seconds in 0.3% NaCl to remove remaining adhering soil, snap-frozen in liquid nitrogen and stored at −80 °C. Homogenized root powder was freeze-dried and the carbon isotopic composition of the root tissue was measured by stable isotope ratio mass spectrometry (DELTAplusCP, Thermo Fisher Scientific, US).

### 4.3. Root RNA Sequencing

Roots from the topsoil (0–20 cm, D1) were assessed by RNAseq and gene expression analysis. Total RNA was extracted from an aliquot of 50 mg homogenized root powder using the NucleoSpin RNA Plant kit (Macherey-Nagel, Germany) according to the manufacturer’s protocol. The total RNA was treated with RNase-free DNase set (Qiagen, Germany). RNA quality and quantity were checked using a Nanodrop spectrophotometer (Thermo Fisher Scientific, US) and a Bioanalyzer 2100 on a Plant RNA Nano chip (Agilent, US). All RNA samples passed quality checking with RNA integrity numbers (RIN) > 8. For RNA sequencing we used six biological replicates, except for *rth3* treatments in BBCH19/59 with three biological replicates. 

RNA sequencing was conducted at the Genewiz sequencing facility (Leipzig, Germany) with a stranded paired-end 150 bp library design on the Illumina NovaSeq 6000 platform (Illumina, US). Raw reads were processed as described by Ganther et al. (2020) [90]. Briefly, raw read qualities were estimated using ‘FastQC’ [91], and the reads were trimmed and quality-filtered with ‘Trimmomatic’ [92]. The cleaned reads were aligned to the maize B73_v4.47 reference genome [93] with ‘HISAT2’ [94] and quantified with ‘featureCounts’ of the ‘Subread’ package [95]. All following downstream analyses were conducted in R [96]. Significant differences in gene expression were determined using the ‘DESeq2’ package [97]. Correction of *p*-Values for multiple testing was done with the Benjamini-Hochberg adjustment [98]. Genes with an adjusted *p*-Value *p* < 0.05 and an absolute log_2_ fold change LFC > 1 were considered as differentially expressed. Differentially expressed genes were analyzed for their biological functions using gene ontology (GO) annotation. Enriched GO terms were determined with the ‘goseq’ package [99], utilizing the ‘maize-GAMER’ GO annotation by Wimalanathan et al. (2018) [100]. Permutational analysis of variance (PERMANOVA) was performed with the ‘adonis’ function of the ‘vegan’ package [101], using Euclidean distances with 999 permutations. Variance partitioning analysis was performed with the ‘variancePartition’ package [102].

### 4.4. qPCR Arrays

Differential gene expression of selected genes was assessed in roots from the upper (0–20 cm, D1) and lower (20–40 cm, D2) depth of the soil on a 96.96 Dynamic Array chip (Fluidigm). A laboratory study [38] and preliminary RNA sequencing results from the stage BBCH14 showed that genes related to the functional terms nutrient and water transport, plant immunity, and root exudation, were differentially expressed between soil depths and between sand and loam. For the qPCR arrays, we selected 41 genes of these functional categories according to the respective transcript abundances in the two experiments, as well as three reference genes. The corresponding primers are listed in Table S9. Primers were used for pre-amplification of PCR products and the qRT-PCR reaction in the chip.

First-strand cDNA was synthesized from 750 ng RNA using the SuperScript IV First-Strand Synthesis System (Thermo Fisher Scientific) according to the manufacturer’s protocol. First-strand cDNA was pre-amplified according to the manufacturer’s instructions using MyTaq DNA Polymerase (Bioline) and the selected specific primers. For every reaction were added 2 µL of the 5× MyTaq Reaction Buffer, 0.6 µL of MgCl_2_ (25 mM), 0.2 µL of dNTPs (10 mM), 0.2 µL of MyTaq DNA Polymerase, 4 µL of nuclease-free water and 2 µL of 5× pooled specific primers (250 nM). Pre-amplification conditions were 5 min at 95 °C followed by 18 cycles at 95 °C for 10 s, 60 °C for 1 min and 72 °C for 15 s. The pre-amplified cDNA was treated with Exonuclease I (New England BioLabs) according to the manufacturer’s instructions, and quality was checked by real-time qRT-PCR using iQ SYBR Green Supermix and the iQ5 real-time PCR detection system (Bio-Rad). The qRT-PCR conditions were 10 min at 95 °C followed by 40 cycles at 95 °C for 15 s and 60 °C for 1 min. The specificity of each PCR amplification procedure was verified by melt-curve analysis of the PCR product with a heat dissociation protocol (from 60 to 95 °C). The qPCR products were diluted at 1/40 with TE buffer.

The gene expression was analyzed by qRT-PCR using SsoFast EvaGreen Supermix (Bio-Rad) and the 96.96 Dynamic Array IFC chip for Gene Expression and the BioMark Systems for Genetic Analysis (Fluidigm, US). For each assay, a mix composed of 2.5 µL of 25 µM of each forward and reverse primer and 2.5 µL of the 2× Loading Reagent was loaded into the assay inlets of the array. Into the sample inlets, 5 µL of the solution containing 2 µL of pre-amplified cDNA and 3 µL of a mix composed of 1× SsoFast EvaGreen Supermix (Bio-Rad), 1× loading reagent and 1× ROX were loaded. The cycling program consisted of 10 min at 95 °C followed by 40 cycles at 95 °C for 15 s and 60 °C for 1 min. Gene expression values were normalized to the mean of the three reference genes (*GAPDH*, *Actin1*, and *EF-1α*) and analyzed with the ΔΔCT method [103]. Significant differences between treatments were determined by ANOVA and Tukey’s HSD test and corrected for multiple testing by the Benjamini-Hochberg method (*p* < 0.05).

### 4.5. Shoot and Leaf Elemental Contents

For this study, we assessed the shoot and leaf ionome instead of the root ionome for several reasons. When investigating roots from native environments, traces of remaining soil particles on roots may lead to biased elemental concentrations; whereas on the over hand, extensive root washing may lead to leakage of nutrients, especially for root systems that are not fully intact, such as in this sampling strategy that required cutting the root system within a defined soil volume. 

For this reason, we determined nutrient concentrations of the macronutrients N, P, K, Mg, Ca and S, and micronutrients B, Mn, Fe and Zn in the youngest leaf (YL) for momentary nutrition status, and in the remaining shoot (RS) plus YL for whole shoot nutrient content, in order to evaluate how plant nutrition is affected by the growth stage, substrates and the presence of root hairs. Of note, Vetterlein et al. (2022) report separately on N, P and K uptake in this experiment [39]; these data are duplicated here so that the reader can cross-relate their levels to those of other mineral nutrients. Whole shoot/leaf material was oven-dried at 65 °C for 48 h, milled and homogenized. An aliquot of 30 mg homogenized powder was digested in HNO_3_ (67–69%, Bernd Kraft, Germany) in a high-performance microwave reactor (UltraClave IV, MLS, Selftec, Germany). After digestion, samples were transferred to centrifuge tubes and diluted with de-ionized water. Plant elemental composition was measured using an Inductively Coupled Plasma Optical Emission Spectrometer (ICP-OES, iCAP 7400 OES Duo spectrometer with Qtegra Software, Thermo Fisher Scientific, US). 

## 5. Conclusions

Our results demonstrated that root hairs do play a role in the maintenance of maize growth, while exerting relatively minor differences on transcriptomic level that were highly consistent with laboratory data. That the root hairs mattered for maize growth, supports their potential role in the development of nutrient-efficient maize [46] and further research is necessary to relate the root hair functioning [26] to the observed losses in maize productivity and growth efficiency. We also showed that during their development, maize plants differ in their ability to acquire nutrients, allocate resources and express genes, and that the growth stage determines how strong the impact of the substrate is. These observations call for an integrated plant-soil analysis—targeting also rhizosphere properties, nutrient availabilities, microbiomes and root exudates. The scope of this study was limited to one single growth season, thus not taking year-to-year variations in maize and its rhizosphere into account. As the next step forward, ongoing and future work will consider the performance of maize in the same experimental field setup in the next five consecutive years.

## Data Availability

RNAseq datasets were deposited to the NCBI Short Read Archive (SRA) under the BioProject PRJNA798863. Other datasets generated during and/or analyzed during the current study are available from the corresponding author on reasonable request.

## Supplementary Materials

The following supporting information can be downloaded at: https://www.mdpi.com/article/10.3390/plants11212883/s1, Figure S1. Above-ground daily average temperature and precipitation data for the months May to August at the maize sampling site in Bad Lauchstädt, Germany; Figure S2. Recently incorporated carbon from air (^13^C atomic %) in the shoot remainder; Figure S3. Mineral element concentration (mg/g plant dry weight) in the remaining shoot (RS); Figure S4. Comparison of mobile element concentration in the youngest leaf (YL) and the remaining shoot (RS) for mobile elements N, P, K, and Mg; Figure S5. Mineral nutrient transporter expression in maize roots over time and substrate; Figure S6. Root plasma membrane aquaporin expression in relation to plant age and substrate; Figure S7. Real-time quantitative reverse transcription polymerase chain reaction (qRT-PCR) confirmation of differential gene expression; Figure S8. Differential gene expression between root hairless rth3 and wild type maize in different experiments; Table S1. Effect of substrate and maize genotype on the concentrations of macro- and micronutrients in maize shoot; Table S2. Effect of substrate and maize genotype on the total amounts of macro- and micronutrients in maize shoot; Table S3: Expression of mineral element transporters, acid phosphatases and plasma membrane aquaporins by stage, substrate, and genotype (file Table S3.xlsx); Table S4. Numbers of differentially expressed genes between the treatments; Table S5. Differentially expressed genes by stage, substrate, and genotype (file Table S5.xlsx); Table S6. Enriched GO terms for differentially expressed genes by stage and substrate (file Table S6.xlsx); Table S7. qPCR gene expression in relation to stage, substrate and depth (file Table S7.xlsx); Table S8. Differentially expressed genes (DEG) by root hair genotype (*rth3* vs WT) in three independent experiments (file Table S8.xlsx), Table S9. Primers for RT-qPCR analysis.
