# Supplementary material for: Plant Age and Soil Texture Rather Than the Presence of Root Hairs Cause Differences in Maize Resource Allocation and Root Gene Expression in the Field
Source: Plants (Basel). 2022 Oct 28;11(21):2883. doi: 10.3390/plants11212883 (PMC9657941; doi:10.3390/plants11212883)
Supplement: Supplementary file 1 [file plants-11-02883-s001.zip › Supplementary Figures and Tables.pdf]

Plant age and soil texture rather than the presence of root hairs cause differences in maize resource allocation and root gene expression in the field

Plants

Minh Ganther, Eva Lippold, Manuela Désirée Bienert, Marie-Lara Bouffaud, Mario Bauer, Louis Baumann, Gerd Patrick Bienert, Doris Vetterlein, Anna Heintz-Buschart, Mika Tapio Tarkka*

*Corresponding Author: mika.tarkka@ufz.de (Helmholtz-Institute for Environmental Research - UFZ, Halle, Germany)

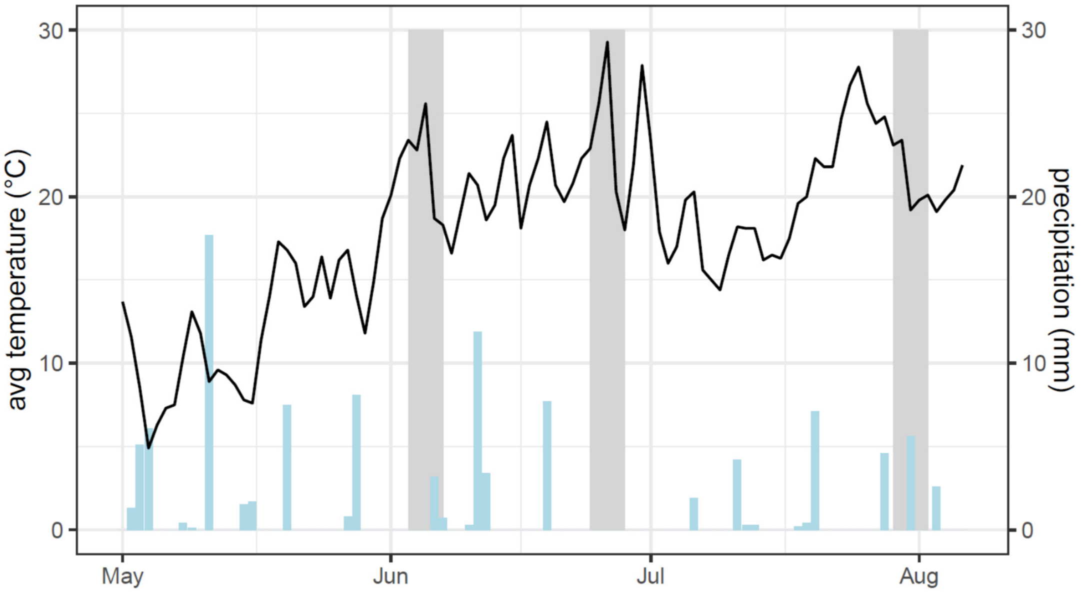

Figure S1. Above-ground daily average temperature and precipitation data for the months May to August at the maize sampling site in Bad Lauchstädt, Germany. Sampling time points (BBCH14: first week of June, BBCH19: last week of June, BBCH59: last week of July) are highlighted with grey bars. Weather data were collected from the DWD Open Data archive (opendata.dwd.de/climate_environment/CDC/observations_germany/climate/ - Location ID 02878)

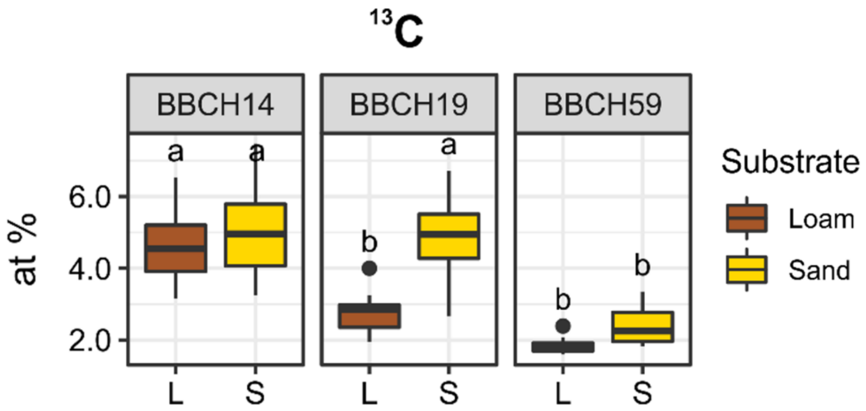

Figure S2. Recently incorporated carbon from air (^{13}C atomic %) in the shoot remainder. Differences between treatments ($P<0.05$) across all stages according to ANOVA and Tukey's HSD test are indicated by different letters. L: loam, S: sand

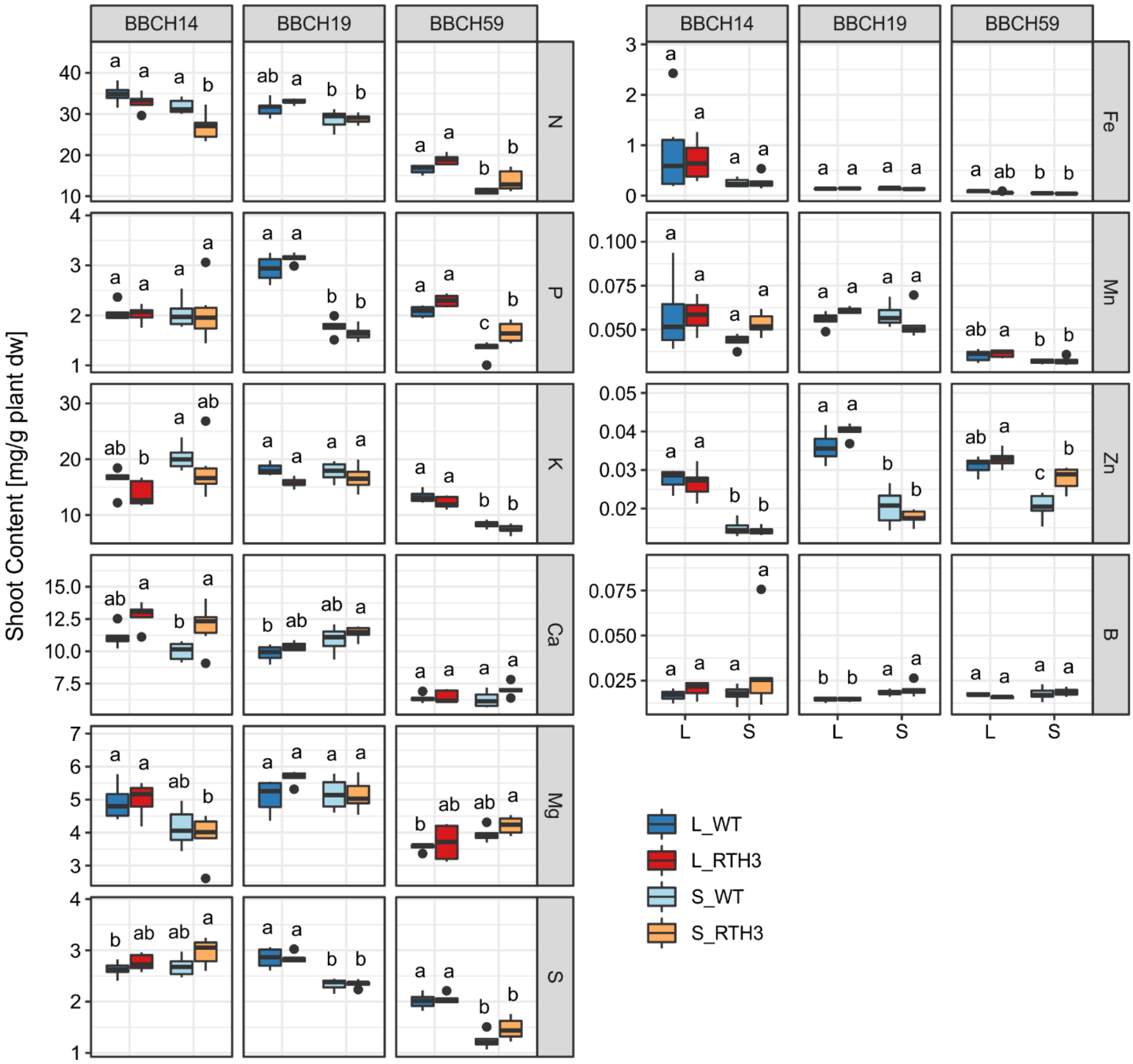

Figure S3. Mineral element concentration (mg/g plant dry weight) in the remaining shoot (RS). The amounts of the mineral nutrients are given by mg per g plant dry weight (dw). Note the different scales for the different mineral nutrients. Differences between treatments ($P<0.05$) within one stage according to ANOVA and Tukey's HSD test are indicated by different letters. L, loam: S, sand; WT, wild type; RTH3, root hair mutant

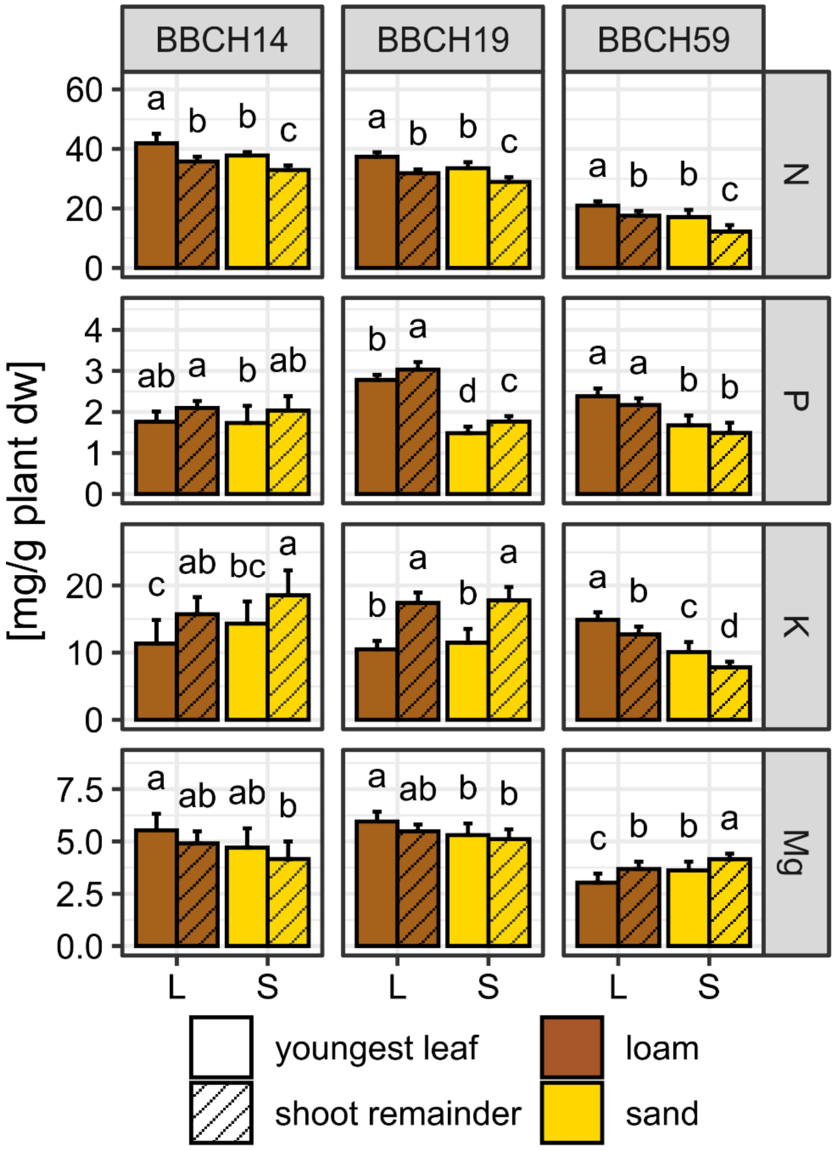

Figure S4. Comparison of mobile element concentration in the youngest leaf (YL) and the remaining shoot (RS) for mobile elements N, P, K, and Mg. The amounts of the mineral nutrients are given in mg/g plant dry weight (dw). Note the different scales for the different mineral nutrients. Differences between treatments (P<0.05) within one stage according to ANOVA and Tukey's HSD test are indicated by different letters. L, loam: S, sand

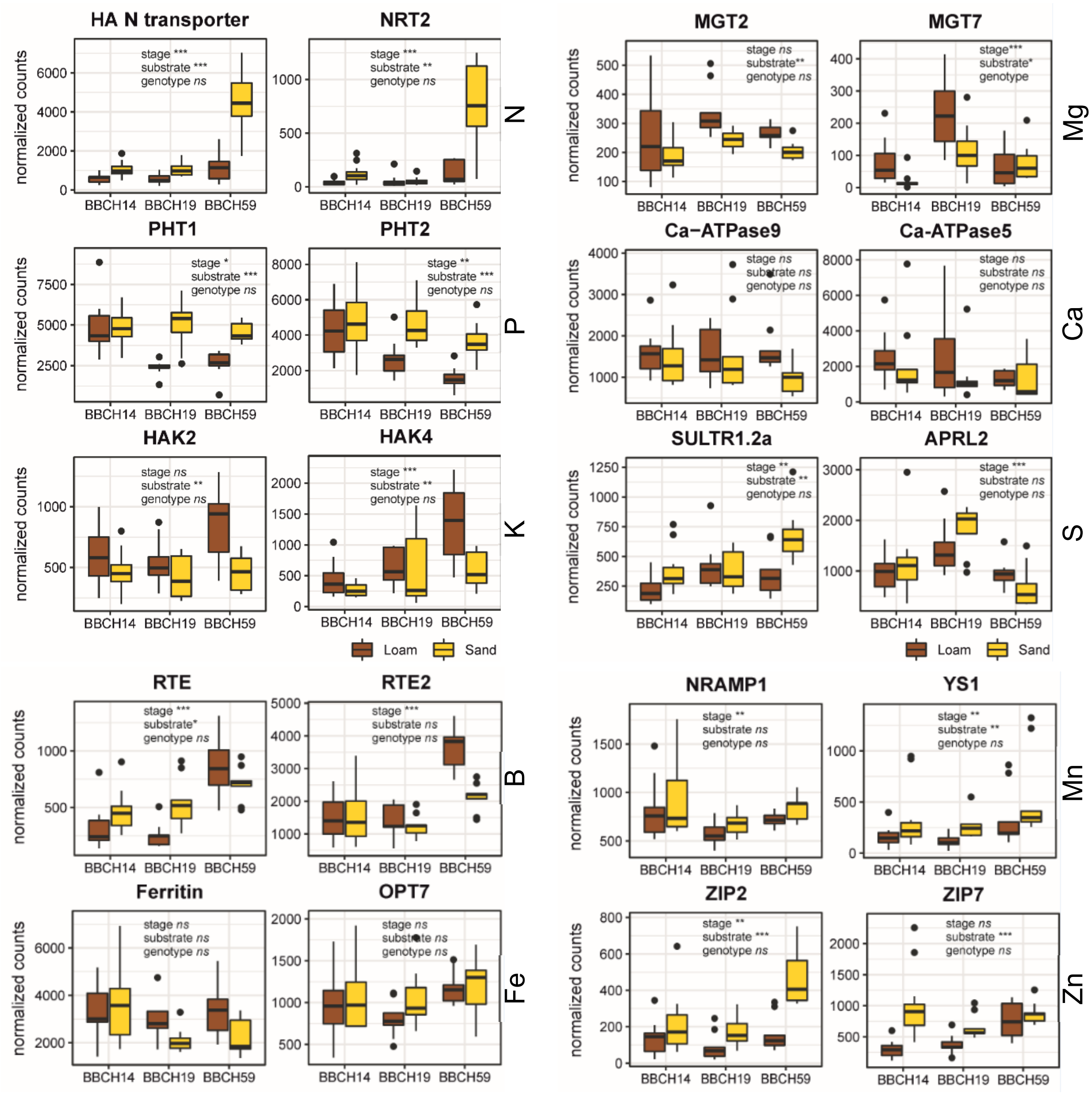

Figure S5. Mineral nutrient transporter expression in maize roots over time and substrate. Treatments with different letters are significantly different (ANOVA, Tukey's HSD test, P<0.05). Asterisks indicate significant impact of grouping factor (Likelihood ratio test, *** P<0.001, ** P<0.01, * P<0.05, ns not significant). L, loam; S, sand. The presence of root hairs did not affect the transporter gene expression levels.

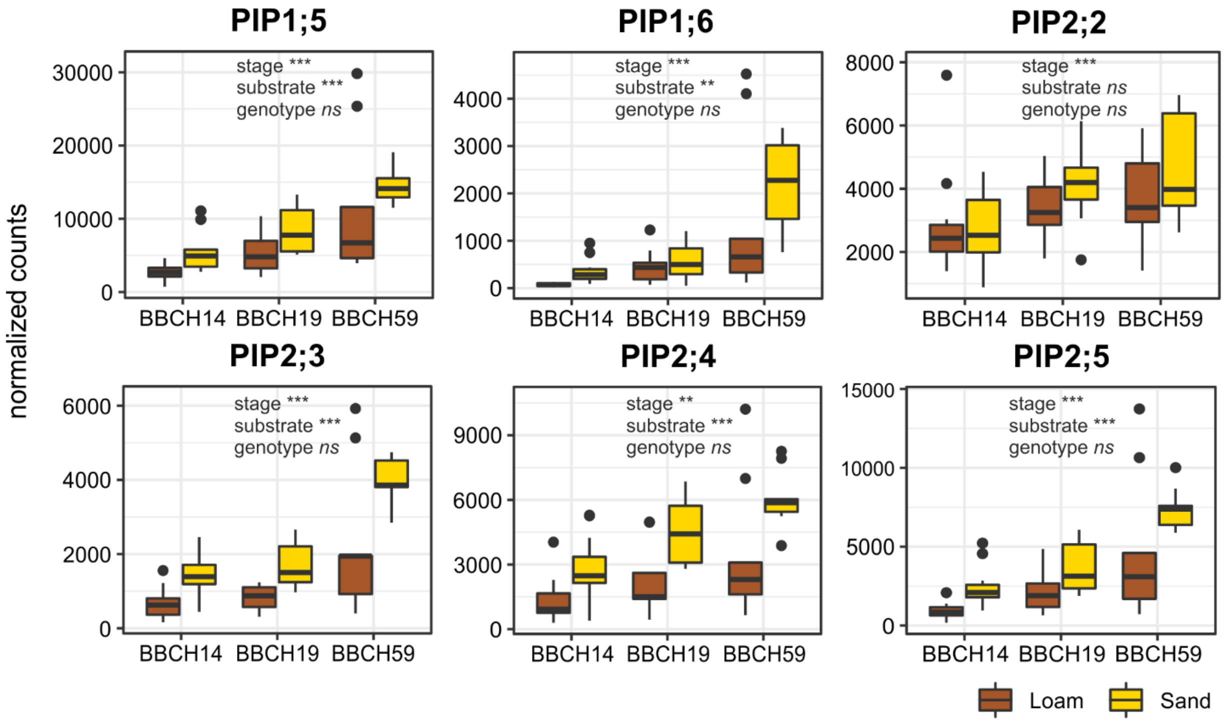

Figure S6. Root plasma membrane aquaporin expression in relation to plant age and substrate. Root gene expression levels (normalized counts) for selected related aquaporin genes. RNA sequencing analysis of mRNA in roots of maize. Data are represented as DESeq2 normalized counts. Due to low variation between the *rth3* and wild type maize the data from the two genotypes were combined for this analysis. Asterisks indicate significance of the respective experimental factor (Likelihood ratio test, *** P<0.001, ** P<0.01, * P<0.05, ns not significant)

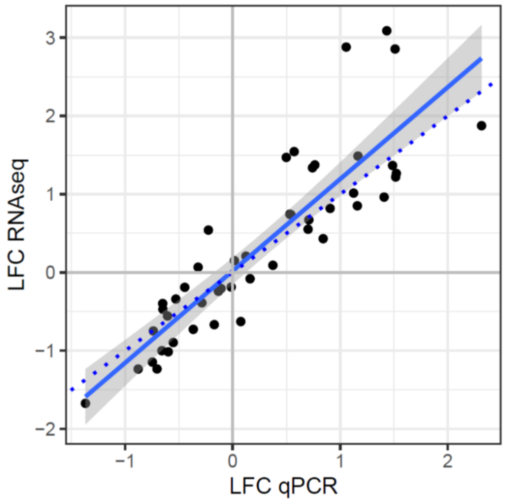

Figure S7. Real-time quantitative reverse transcription polymerase chain reaction (qRT-PCR) confirmation of differential gene expression. Comparison of log_2 fold changes (LFC) for RNAseq and qRT-PCR data. The plotted data points were calculated from the sand vs loam contrast for 41 surveyed qPCR array genes. The blue solid line represents a linear regression model, whereas the dotted line represents the ideal outcome with equal values for RNAseq and qPCR data. LFC values from RNAseq data were calculated with the DESeq package (Wald test), and LFC values from qPCR data were derived by using the ΔΔCT method. GAPDH, Actin1 and EF-1a served as reference genes of qPCR analysis

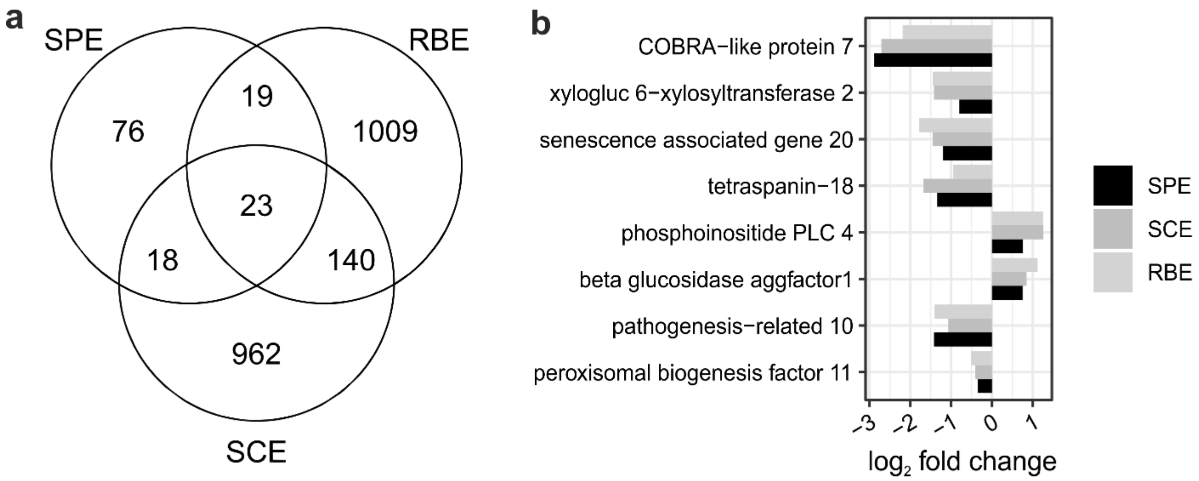

Figure S8. Differential gene expression between root hairless *rth3* and wild type maize in different experiments. Differential expression (RNAseq data) between *rth3* and wild type was assessed for three different experiments. SPE: field-scale, developmental stage: BBCH14-BBCH59, mix of total roots from 0-20 cm topsoil. SCE: lab-scale, soil column, developmental stage: BBCH14, total roots from 1.4 cm soil slices along the soil column. RBE: lab-scale, rhizobox, developmental stage: 10 days after germination, primary roots. (a) Venn diagram showing the overlap of differentially expressed genes by root hair formation in the three experiments. (b) Comparison of expression level changes (log_2 fold changes) of eight genes between the three experiments (taken from the overlap in a)). The displayed genes related to processes in cell wall organization, signaling and stress response

Table S1. Effect of substrate and maize genotype on the concentrations of macro- and micronutrients in maize shoot. Significance of the experimental factor was determined by ANOVA and Tukey's HSD test. Level of significance: *** P<0.001, ** P<0.01, * P<0.05, *ns* not significant

		Effect significance during growth stages		
**Element**	**Factor**	**BBCH14**	**BBCH19**	**BBCH59**
Macronutrients				
Ca	Substrate	ns	**	ns
Ca	Genotype	**	ns	*
Ca	Substrate:Genotype	ns	ns	ns
K	Substrate	**	ns	***
K	Genotype	ns	*	*
K	Substrate:Genotype	ns	ns	ns
Mg	Substrate	**	ns	**
Mg	Genotype	ns	ns	ns
Mg	Substrate:Genotype	ns	ns	ns
N	Substrate	***	***	***
N	Genotype	**	ns	**
N	Substrate:Genotype	ns	ns	ns
P	Substrate	ns	***	***
P	Genotype	ns	ns	***
P	Substrate:Genotype	ns	*	ns
S	Substrate	ns	***	***
S	Genotype	*	ns	*
S	Substrate:Genotype	ns	ns	ns
Micronutrients				
B	Substrate	ns	***	ns
B	Genotype	ns	ns	ns
B	Substrate:Genotype	ns	ns	ns
Fe	Substrate	*	ns	***
Fe	Genotype	ns	ns	*
Fe	Substrate:Genotype	ns	ns	ns
Mn	Substrate	ns	ns	**
Mn	Genotype	ns	ns	ns
Mn	Substrate:Genotype	ns	*	ns
Zn	Substrate	***	***	***
Zn	Genotype	ns	ns	***
Zn	Substrate:Genotype	ns	*	*

Table S2. Effect of substrate and maize genotype on the total amounts of macro- and micronutrients in maize shoot. Significance of the experimental factor was determined by ANOVA and Tukey's HSD test. Level of significance: *** P<0.001, ** P<0.01, * P<0.05, *ns* not significant

		Effect significance during growth stages		
**Element**	**Factor**	**BBCH14**	**BBCH19**	**BBCH59**
Macronutrients				
Ca	substrate	ns	***	**
Ca	genotype	**	***	***
Ca	substrate:genotype	ns	ns	ns
K	substrate	ns	***	***
K	genotype	***	***	***
K	substrate:genotype	ns	ns	*
Mg	substrate	*	***	***
Mg	genotype	***	***	***
Mg	substrate:genotype	ns	ns	ns
N	substrate	ns	***	***
N	genotype	***	***	**
N	substrate:genotype	ns	*	ns
P	substrate	ns	***	***
P	genotype	**	***	**
P	substrate:genotype	ns	*	*
S	substrate	ns	***	***
S	genotype	**	***	**
S	substrate:genotype	ns	*	*
Micronutrients				
B	substrate	ns	***	**
B	genotype	ns	***	***
B	substrate:genotype	ns	ns	ns
Fe	substrate	*	***	***
Fe	genotype	ns	***	***
Fe	substrate:genotype	ns	ns	*
Mn	substrate	*	***	***
Mn	genotype	**	***	***
Mn	substrate:genotype	ns	ns	ns
Zn	substrate	***	***	***
Zn	genotype	***	***	**
Zn	substrate:genotype	*	*	*

Table S3. Expression of mineral element transporters, acid phosphatases and plasma membrane aquaporins by stage, substrate, and genotype (file Table S3.xlsx)

Table S4. Numbers of differentially expressed genes between the treatments. Differential gene expression was identified by Wald tests in DESeq2. Multiple testing correction was performed using Benjamini-Hochberg adjustment of p-values. Genes expression differences with P<0.05 and a two-fold difference in gene expression were considered as significant. Treatments refer to a contrast of condition1 vs condition 2 (baseline). LFC, log_2 fold change

treatment	up-regulated	down-regulated
BBCH19 vs BBCH14	789	752
BBCH59 vs BBCH19	743	586
BBCH59 vs BBCH14	1187	1350
sand vs loam	780	568
rth3 vs WT	76	21
BBCH14: sand vs loam	283	390
BBCH19: sand vs loam	800	444
BBCH59: sand vs loam	2218	997

Table S5. Differentially expressed genes by stage, substrate, and genotype (file Table S5.xlsx)

Table S6. Enriched GO terms for differentially expressed genes by stage and substrate (file Table S6.xlsx)

Table S7. qPCR gene expression in relation to stage, substrate and depth (file Table S7.xlsx)

Table S8. Differentially expressed genes (DEG) by root hair genotype (*rth3* vs WT) in three independent experiments (file Table S8.xlsx)

Table S9. Primers for RT-qPCR analysis. Primers were constructed for maize genes associated with mineral nutrient or water acquisition, root exudation, stress and immunity. Selection was based on their expression level in the field and laboratory experiments and differential gene expression between the substrates loam and sand, determined by RNA sequencing

gene_id	primer sequence (5' - 3')	direction	description
Zm00001d033862	GTCAAGGGCGTGCTCATC	forward	ACC synthase6
	AAGTCCACCAGCATCTCCAG	reverse	
Zm00001d003712	ACCACCAACGACGACGAATA	forward	ABA stress ripening3
	CGGGAGGAGCCGTACTCT	reverse	
Zm00001d019406	TGAAAGGGTACAGCAACTCCG	forward	Acidic endochitinase
	ACCGACCCAGCTATCAGGAA	reverse	
Zm00001d026102	CAGCAGCTCACCAAACCACT	forward	Al-activated malate transporter 10
	ACGAGAAGAGACACACCACC	reverse	
Zm00001d003749	AATCTGTCGGTACCCTTGCG	forward	Al-activated malate transporter 8
	AGCGTTGTTCTCATGGTCGT	reverse	
Zm00001d048709	CTGAGGAGGCTGGAGGAGTAT	forward	benzoxazinless1
	TTTATTGGTCGGTCGTCTTCTC	reverse	
Zm00001d043988	CAGAGCAGCGACATCCAGAC	forward	Chitinase chem5
	GTTGTCCCACAGGTAGTCGG	reverse	
Zm00001d017292	ATCCCCCTTTCTGCTCCTGT	forward	Defensin-like protein 6
	CTCTGTACTCACTGGCTCGC	reverse	
Zm00001d035129	GATGCACAGGGAGGGATCT	forward	Disease resistance RPP13-like 4
	TGCCTCGTCTTATTGGTCTCAG	reverse	
Zm00001d025050	AGAAACCGGTCACATTAGCC	forward	Ethylene receptor homolog2
	TGAGAACCGTCAGGCTCTTT	reverse	
Zm00001d002762	GACGACATGGTGGTGTTCGG	forward	Ethylene-responsive TF2
	GGAGTAGTAGGAGTCGGGGG	reverse	
Zm00001d042611	GCGGCCTGGTGTTCTTTTC	forward	Gibberellin 20-oxidase3
	TGAACTATCCATCGTCCATAGG	reverse	
Zm00001d041656	CACCACCAACGCCAGTAGTA	forward	Jasmonate-induced protein
	TTGACAGCATCGACCGACAT	reverse	
Zm00001d033623	GCACGAGACGGACCATGTAT	forward	Lipoxygenase
	GGAGCACTGCGAAACGACTA	reverse	
Zm00001d035115	TCAATGGTCCTTGTTGCCGT	forward	MATE1
	GCCGTCCCTATCCTCCAAAA	reverse	
Zm00001d028815	CAGCTGGACTGTTGAGATCG	forward	Pathogenesis-related protein 10
	GTGTGCCAGTCCATCACG	reverse	
Zm00001d048947	ACCTTCACTTTTCCTCCTGCT	forward	Pathogenesis-related protein3
	ACACCGTCTCGTAGTCCAGG	reverse	
Zm00001d011080	TGATCCTCAAGGACAAGCCG	forward	Protease inhibitor; Proteinase inhibitor
	AAGATGCGGACACGGTTAGG	reverse	
Zm00001d012660	CCGAAGTGCTCTATGCGAGT	forward	NPR1
	TGCAACCGAAGGTCTCAGTG	reverse	
Zm00001d027854	TGTGGCTATGCGGTCCTATTG	forward	sucrose transporter1
	CAGTGCAGGAGGTGGAAGGA	reverse	
Zm00001d041067	TTGAGACAGCAGAGGACACT	forward	SWEET13c
	CGAATGCCAACAGTTGCAGA	reverse	
Zm00001d015905	GCGCGACTTGTTATTGGTGT	forward	SWEET4a
	GAGAGACCGTGTGATGCGAT	reverse	
Zm00001d041111	AGGAGTTCTTCTTCGTCGCA	forward	transporter of mugineic acid1

	GCACCAAGAAAACCAGCGTA	reverse	
Zm00001d051872	CACGTGGTCATCATCAGGG	forward	PIP1;5
	CGTATGCTGCATGGTTGCT	reverse	
Zm00001d051174	AGTACGTGCTGAGAGCCAGC	forward	PIP2;3
	CGTACGTATCTACACTTGGATCGAT	reverse	
Zm00001d019565	TTTAAGGTGAACGGAGAAGGAGA	forward	PIP2;6
	GAAAGCTACTGCTGCTGTGGAT	reverse	
Zm00001d032850	CATTGTCACGCTCGTCATCT	forward	PHT2
	GGTGGAGTTGAAGTGGTCGT	reverse	
Zm00001d031875	AGTCCAAGGGCAAATCCCTG	forward	PHT7
	GCACTGTGCGGTTGTTGTAG	reverse	
Zm00001d004308	TACTTCGGCACCAACGAAGC	forward	PHT12
	CTCTTGTTGGCGTACTCGGA	reverse	
Zm00001d038252	CGACGGCTGAATTCTGAACC	forward	KCH2
	GGCGATCAGGTAGTACAGGC	reverse	
Zm00001d037289	ATGTTAAGTCGCCCCAGGTC	forward	ZORK
	GCGAACATCCCACTGGCTA	reverse	
Zm00001d003859	AGGTGCTCAAAGTGGGGATG	forward	HAK20
	CTGGCTGCCAAGCAAGTACA	reverse	
Zm00001d017249	TCTTCCTCTTCCAGTGGGCT	forward	AMT2
	GCGGAGTAGATGAGGTACGC	reverse	
Zm00001d012261	CATACCGGACTGGCTGAACA	forward	AMT7
	GGCCCACTTCTTCTTCACGA	reverse	
Zm00001d054057	CCTATGGGCGGCATCATGT	forward	NRT2
	CCAGATGTTCCAGAGGCGAG	reverse	
Zm00001d014976	CGCATGGTAGCTCGCTGGA	forward	NRT3
	GGATGGTCTTGGCCTTGTGC	reverse	
Zm00001d011679	GCATCGTCCCGTTCGTCTC	forward	NRT5
	CCGTCTCCGTCTTGTACTTGG	reverse	
Zm00001d026590	TGACGTTTCAGGTGTTCTCTG	forward	Auxin response factor 8
	GCAACTGTATTTTCGTGGTC	reverse	
Zm00001d032883	ACATCACGTACACCAGATTGC	forward	Expansin-A11
	GTCTGCACATTGTTGGTAGA	reverse	
Zm00001d003368	GGCTCCCATGAAAACGCAAA	forward	Kinesin KP1
	CCACTGGAACGAGATGCTGC	reverse	
Zm00001d014507	CGACGAGCCTTTCAGTGAGT	forward	Auxin response factor 16
	AGACTGGACTCTACCTCGCT	reverse	
Zm00001d010159	GCCCTGCTGTATGAAATGGA	forward	Actin1
	AAAGGAACCAGCTAAAAGCAAAC	reverse	
Zm00001d049641	TTGTTTCCCTTCCTGCTACC	forward	GAPDH
	AAACTGCAACCTCACCACAAG	reverse	
Zm00001d046449	TGGGCCTACTGGTCTTACTACTGA	forward	EF1-alpha
	ACATACCCACGCTTCAGATCCT	reverse	
